# Health Care Employees’ Perceptions of the Use of Artificial Intelligence Applications: Survey Study

**DOI:** 10.2196/17620

**Published:** 2020-05-14

**Authors:** Rana Abdullah, Bahjat Fakieh

**Affiliations:** 1 Information Systems Department King Abdulaziz University Jeddah Saudi Arabia

**Keywords:** artificial intelligence, employees, healthcare sector, perception, Saudi Arabia

## Abstract

**Background:**

The advancement of health care information technology and the emergence of artificial intelligence has yielded tools to improve the quality of various health care processes. Few studies have investigated employee perceptions of artificial intelligence implementation in Saudi Arabia and the Arabian world. In addition, limited studies investigated the effect of employee knowledge and job title on the perception of artificial intelligence implementation in the workplace.

**Objective:**

The aim of this study was to explore health care employee perceptions and attitudes toward the implementation of artificial intelligence technologies in health care institutions in Saudi Arabia.

**Methods:**

An online questionnaire was published, and responses were collected from 250 employees, including doctors, nurses, and technicians at 4 of the largest hospitals in Riyadh, Saudi Arabia.

**Results:**

The results of this study showed that 3.11 of 4 respondents feared artificial intelligence would replace employees and had a general lack of knowledge regarding artificial intelligence. In addition, most respondents were unaware of the advantages and most common challenges to artificial intelligence applications in the health sector, indicating a need for training. The results also showed that technicians were the most frequently impacted by artificial intelligence applications due to the nature of their jobs, which do not require much direct human interaction.

**Conclusions:**

The Saudi health care sector presents an advantageous market potential that should be attractive to researchers and developers of artificial intelligence solutions.

## Introduction

### Overview

Recently, health care systems in several countries have begun to rely on storage of patient information to provide the best quality of health care. Due to rapid technological developments, health care information technology solutions provide the capacity to store enormous volumes of patient data; however, appropriate utilization of this data is essential to enhance health care quality, improve decision making, and reduce costs [1,2]. Over the last decade, artificial intelligence (AI) has provided significant advancements in this regard [3].

Artificial intelligence technologies were developed to offer practical benefits in different areas including health care applications [4,5]. A common feature of AI is the replication of human intellectual functions. From the health care perspective, AI brings a “paradigm shift to health care, powered by increasing availability of health care data and rapid progress of analytics techniques” [6].

Despite its promise, health sector employees have mixed attitudes and feelings regarding the implementation of AI technologies [7,8]. This study investigated the attitudes and perceptions of the emergence of AI technologies among health care employees in Saudi Arabia.

### Related Studies

Sarwar et al [9] used a questionnaire distributed to 487 pathologist respondents from 54 countries to explore perspectives of AI implementation in clinical practice. Their findings revealed generally positive attitudes toward AI, with approximately 75% reporting excitement or interest in AI as a diagnostic tool for improving quality and efficiency in pathology workflows. About 80% of the participants predicted the introduction of AI technology into the pathology laboratory in the coming years [9].

According to Brougham and Haar [10], futurists expect that nearly one-third of all existing jobs could be replaced by AI, smart technology algorithms (STARA), and robotics by the year 2025. There is limited information, however, about how employees perceive these technological innovations within the scope of their own careers and how they are being prepared for these possible changes. STARA awareness was created as a new measure for this research, capturing the degree to which employees feel their job might be replaced by technology. As career progression and associated technology knowledge increase with age, age was also tested as a STARA moderator. By employing a mixed methods approach with 120 employees, STARA awareness was tested on a range of jobs and well-being. STARA awareness was inversely correlated with career satisfaction and organizational commitment, and directly correlated with depression, cynicism, and turnover intentions [10].

Alamanova [11] investigated the perceptions of AI among human resource (HR) professionals in the fields of leadership, consultancy, and recruitment. The results showed that HR professionals have different feelings about AI than they do about other new technologies, as they were excited about reducing manual workloads while remaining cautious about adding excessive functionality to computing machines. In addition to electronic human resource management applications, HR professionals had concerns regarding technology interfaces and pricing [11].

Zande [12] explored workforce perspectives on Robotic Process Automation (RPA) implementation by conducting 8 interviews with staff whose jobs are automated by RPA. The study concluded that employees perceived the implementation of RPA as positive before and after its implementation. Because of the simple nature of the automated processes, employees believed that RPA implantation reduced their workload. The employees also expected their jobs to become more diverse and interesting. They continued to feel positive after implementation while also expressing concerns about the occurrence of process errors and how to handle those errors when the availability of technology expertise is limited [12].

Maskara et al [13] investigated current and future applications as well as employee acceptance of AI in the medical field. The sample included 73 cardiologists, dentists, ophthalmologists, and surgeons. Phone-based and face-to-face interviews were conducted to understand respondents’ perception and awareness of AI solutions. Most respondents were aware of AI interventions in use in their field and some were already harnessing AI themselves; however, while doctors were aware of the advantages of AI advantages, they also perceived disadvantages in the high cost and lack of human touch [14].

Oh et al [8] explored AI awareness among doctors and assessed their attitudes toward medical AI applications. An online questionnaire conducted and distributed to 669 participants showed that doctors have positive attitudes toward AI implementation in the medical field. Most of the surveyed physicians assumed that their roles will not be replaced by AI [8].

Van Ittersum et al [13] sought to understand technology acceptance through review study and qualitative model development. They found that several variables influence technology acceptance, including the technology itself, characteristics of the individual user, and features of the organization of technologies used in the work environment. Individual user characteristics and technology characteristics interact to influence acceptance in terms of attitudes, intentions, and behaviors. Other variables such as technophobia, knowledge, or prior experience can be changed through exposure or training and instruction. Companies therefore have an opportunity to influence acceptance. A similar logic applies to organizational user characteristics. Opportunities to influence acceptance are provided when the variables affecting that acceptance are understood, such as ease of use, complexity, and fun/enjoyment, which can also be influenced through marketing activities. Other factors such as privacy, risk, and compatibility can be considered during the design process to maximize acceptance by at least some user groups [13].

Although these studies concentrated on the employee perceptions of AI implementation in their fields, the influence of different job types was not considered, nor did these studies consider respondents’ knowledge of AI, although such knowledge could significantly influence perception. Finally, there is a clear lack of research on this subject in the Arabian and Saudi context despite the revolution that this area is witnessing in this field. This study was intended to fill this gap and find answers to the following questions:

1. What is the level of employees’ knowledge about AI in the health care sector?
2. What is the perception of AI implementation among employees in the health care sector in Saudi Arabia?
3. Does job type influences perceptions of AI implementation of?

Table 1 summarizes prior studies on the subject.

**Table 1:** Summary of related studies.

Author	Year	Country	Field	Study type	Sample size	Main results
Alamanova [11]	2018	Estonia	Human resources	Qualitative	5	Varying feelings about AI^a^ Focus on technology price and interface
Zande [12]	2018	Netherlands	Jobs automated by RPA^b^	Qualitative	8	Positive attitudes toward technology implementation Participants showed little concern
Maskara et al [14]	2017	India	Medical field	Qualitative	73	Most respondents aware of AI interventions Some perceived disadvantages of AI
Oh et al [8]	2019	South Korea	Medical field	Quantitative	669	Positive attitudes toward AI implementation in the medical field Assumption that roles will not be replaced by AI

^a^AI: artificial intelligence.

^b^RPA: robotic process automation.

## Methods

**Figure 1:**
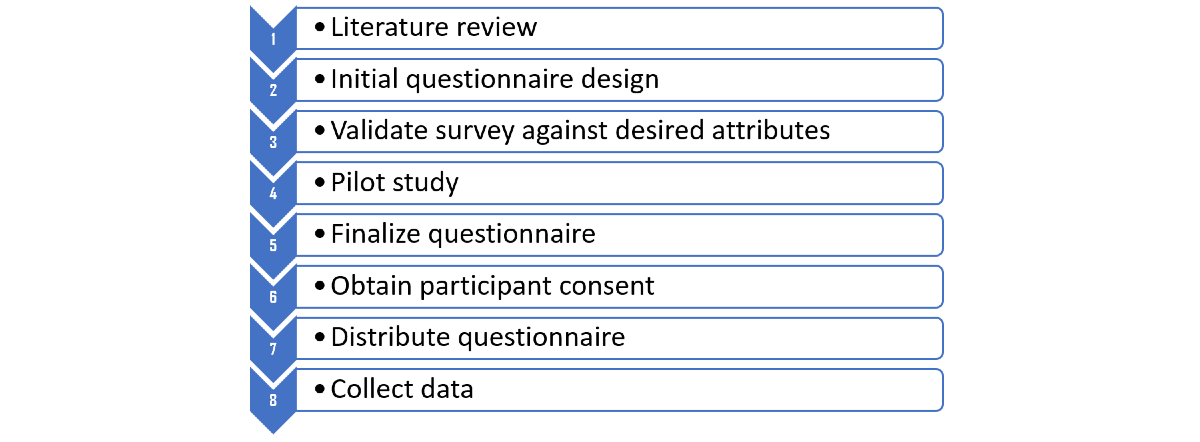
Survey workflow.

This study employed a descriptive analytical method and relied on a quantitative approach to collect primary data. The study was conducted from September 2018 to April 2019 at 4 of the main hospitals in Riyadh, Saudi Arabia. Figure 1 illustrates the study workflow. Each phase of the study will be discussed in detail in the following sections.

### Population and Sample

The study population included all employees in public health care institutions in Saudi Arabia in the years 2018 and 2019, with a total of 300,699 working staff including physicians, nurses, pharmacists, and support staff [15]. The study sample consisted of 250 employees in the 4 largest hospitals in Riyadh, Saudi Arabia, which is home to 618 health institutions [16]. The sample included three types of employee: doctors, nurses, and technicians. Adequate sample size was met by convenience [17] and snowball sampling [18]. We employed Cohen’s formula, which suggests that the effect size is low when the *r* value varies around 0.1, medium when the *r* value varies around 0.3, and large when the *r* value varies more than 0.5 [19]. The power analysis showed that a sample of 250 would provide an 80% chance of detecting correlations of ±0.223 at *P*≤.05.

### Questionnaire

The questionnaire was adapted from Oh et al [8] with revision, including changing the questions from multiple choice to a Likert scale and omitting questions related to pure medicine, as the original questionnaire targeted doctors. The questionnaire in its final form consisted of two main parts. The first part comprised a set of demographic questions to capture gender, age, job type, and level of educational attained. The second part of the questionnaire consisted of three sections. The first section (perceptions of AI) included four items: “I have a good knowledge about AI”, “AI abilities are superior to the experience of humans”, “AI could replace me in my job”, “I have high hopes about AI applications in the health care sector”. The second section (the advantages of using AI) included five items: “AI can speed up the process in health care”; “AI can help reduce medical errors”; “AI can deliver clinically relevant, vast amounts of high-quality data in real time”; “AI has no space-time constraint”; “AI suffers no emotional exhaustion or physical limitation”. The final section (problems for AI application in health care) included five items: “AI cannot be used to provide opinions in unexpected situations”, “AI is not flexible enough to be applied to every patient”, “AI is difficult to apply to controversial subjects”, “AI has low ability to sympathize and consider the emotional well-being of the patient”, “AI was developed by a specialist with little clinical experience in medical practice”.

The questionnaire was validated by introducing it to a panel of experts in the medical and AI fields and circulated to a pilot sample of 15 employees from outside the sample population. Participants at this stage were asked to assess the clarity of the questions. Questionnaire reliability was established through the test-retest method, which is used to test changes when measuring a stable individual characteristic on different occasions [20]. The pilot study was resent to the same pilot sample of 15 employees after a 3-week period.

### Data Analysis

Data were collected, categorized, and coded using Microsoft Excel (Microsoft Corporation) and then analyzed using Statistical Package for the Social Sciences (IBM Corporation). The mean, standard deviation, frequencies, and percentages were calculated for each question, while analysis of variance (ANOVA) was used to test for significant differences between the different demographic variables. Likert scores of 1-2.60 were considered low, 2.61-3.40 were considered moderate, and 3.41-5 were considered high.

## Results

Table 2 shows the distribution of demographic variables among the questionnaire sample. Most of the sample (187/250, 74.8%) were female, as the majority of the sample were nurses (121/250, 48.4%) and between 20 and 40 years old (186/250, 74.8%). Moreover, nearly half of the respondents had a bachelor’s degree (138/250, 55.2%).

**Table 2:** Respondent demographics (N=250).

Demographic and variable		Frequency, n (%)	
**Gender**			
	Male		63 (25.2)
	Female		187 (74.8)
**Age (years)**			
	20-30		132 (52.8)
	31-40		54 (21.6)
	41-50		58 (23.2)
	>50		6 (2.4)
**Job type**			
	Doctor		70 (28)
	Nurse		121 (48.4)
	Technician		59 (23.6)
**Educational level**			
	Diploma		47 (18.8)
	Bachelor		138 (55.2)
	Postgraduate		65 (26)

Table 3 lists the respondents’ answers regarding perception of AI technologies. The overall perception toward AI was moderate, with a mean of 3.01 (SD 1.13). More specifically, the item “AI could replace me in my job” ranked first with a mean of 3.11 (SD 1.17) or moderate on the Likert scale, while the least-applicable item was “I have good knowledge of AI” with a mean of 2.95 (SD 1.14) or moderate on the Likert scale.

**Table 3:** Perceptions of AI (N=250).

Rank	Question		Mean (SD)	*n*	Approximate agree rate	Level
4	I have good knowledge of AI^a^		2.95 (1.14)	185	74%	Moderate
2	AI abilities are superior to human experience		3.01 (1.17)	187	75%	Moderate
1	AI could replace me in my job		3.11 (1.13)	195	78%	Moderate
3	I have high hopes about AI applications in the health care sector		2.96 (1.11)	185	74%	Moderate
N/A^b^		Overall perception of AI	3.01 (1.13)	187	75%	Moderate

^a^AI: artificial intelligence.

^b^N/A: not applicable.

Table 4 lists respondents’ answers regarding the advantages of using AI. Overall, the response was moderate with a mean of 3.36 (SD 1.16). The belief that “AI can speed up the process in health care” ranked first with a high level of acceptance (mean 3.50, SD 1.23). The lowest-ranked item was “AI can deliver clinically relevant, vast amounts of high-quality data in real time” with a mean of 3.24 (SD 1.17), a moderate level on the Likert scale.

**Table 4:** The advantages of using AI (N=250).

Rank	Question	Mean (SD)	*n*	Approximate agree rate	Level
1	AI can speed up the process in health care	3.50 (1.23)	175	70%	High
3	AI can help reduce the number of medical errors	3.36 (1.08)	167	67%	Moderate
5	AI can deliver clinically relevant, vast amounts of high-quality data in real time	3.24 (1.17)	162	65%	Moderate
2	AI has no space-time constraint	3.45 (1.17)	172	69%	High
4	AI has no emotional exhaustion or physical limitation	3.27 (1.16)	162	65%	Moderate
^a^N/A	The advantages of using AI overall perception	3.36 (1.16)	167	67%	Moderate

^a^N/A: not applicable.

Table 5 lists respondents’ perceptions of problems in applying AI in health care. Generally, a moderate response was observed with a mean of 3.37 (SD 1.16). The response “AI is difficult to apply to controversial subjects” ranked first with a high mean of 3.62 (SD 1.17). The lowest-ranked item was a moderate response to “AI cannot be used to provide opinions in unexpected situations” with a mean of 3.20 (SD 1.14).

**Table 5:** The application of AI in health care (N=250).

Rank	Question	Mean (SD)	*n*	Approximate agree rate	Level
5	AI cannot be used to provide opinions in unexpected situations	3.20 (1.14)	160	64%	Moderate
4	AI is not flexible enough to be applied to every patient	3.28 (1.19)	165	66%	Moderate
1	AI is difficult to apply to controversial subjects	3.62 (1.17)	180	72%	High
3	AI has low ability to sympathize and consider the emotional well-being of the patient	3.34 (1.15)	167	67%	Moderate
2	AI was developed by a specialist with little clinical experience in medical practice	3.41 (1.17)	170	68%	High
^a^N/A	Problems regarding the application of AI in health care, overall perception	3.37 (1.16)	167	67%	Moderate

^a^N/A: not applicable.

Table 6 shows the results of the ANOVA test to find the statistically significant differences between the respondents’ answers based on the demographic variables. There was no statistical difference by gender, age, or educational attainment; however, there were significant differences by job type (*P*=.007), with significance defined as .05. Overall, IT technicians in hospitals tended to have the most favorable opinions of AI, followed by doctors.

**Table 6:** ANOVA test of significance.

Variable	F value (*df*)	*P* value
Gender	0.942 (1, 248)	.341
Age	1.51 (3, 246)	.37
Job type	8.680 (2, 247)	.007^a^
Educational level	0.94 (2, 247)	.43

^a^Statistically significant difference.

## Discussion

### Principal Findings

The results of this study showed that employees in the Saudi health care sector have a moderate level of acceptance of AI applications, with most respondents indicating concern that their jobs would be replaced by AI. The results here are inconsistent with the study by Oh et al [8], who indicated that doctors do not believe they will be replaced by AI. The difference between these study findings may be attributed to the low level of knowledge claimed by the respondents in our study versus those in the prior study.

Speeding up health care processes was the main advantage identified by respondents in this study, consistent with prior studies that indicate AI can process a vast amount of data in an accurate, rapid, and efficient way by using complex statistical and computing algorithms [21,22]. In contrast, 163/250 (65%) of respondents did not believe AI can deliver clinically relevant, vast amounts of high-quality data in real time.

The most commonly perceived problem of AI in health care was difficulty in applying AI to controversial subjects and the least commonly identified problem was the inability of AI to provide opinions in unexpected situations, consistent with the findings of Oh et al [8]. The results of this study showed no significant differences in respondents’ answers by gender, age, or educational level. There were significant differences by job type, as technicians were most likely to be exposed to the technological advances of AI, unlike nurses and doctors, who require direct human interaction with patients. This result is consistent with prior studies in which respondents expressed that in the future, computers and robots can do human jobs [23], although doctors feel they are not easily replaced [24].

This study had several limitations: only three job types in the health care sector were polled and the sample included only the largest hospitals in Riyadh. Other limitations include a limited sample size and the descriptive nature of the study. This study could be followed by additional studies including management-level job roles and smaller health care centers. Moreover, experimental studies may provide more realistic and comprehensive results.

### Conclusion

This study assessed employee attitudes toward and perceptions of AI implementation in the health care sector. The study was conducted in 4 hospitals in Riyadh, the capital of Saudi Arabia. The results were mixed between fear of job replacement by AI and a lack of knowledge about AI technologies. Therefore, the results of this study indicate a need for training on the advantages, challenges, and issues surrounding the implementation of AI in health care and the potentials of these technologies to improve health care processes and efficiencies. Training would expand employees’ knowledge of AI and their appreciation of its potential in the health care sector. Governments and universities can play significant roles in advancing health care toward utilizing AI technologies. In addition, the current status of AI use in health care in Saudi Arabia provides an attractive market for AI solution developers.

## Abbreviations

AI: artificial intelligence
ANOVA: analysis of variance
HR: human resources
RPA: robotic process automation
STARA: smart technology algorithms
